# Graph-theoretical prediction of biological modules in quaternary structures of large protein complexes

**DOI:** 10.1093/bioinformatics/btae112

**Published:** 2024-03-06

**Authors:** Florian J Gisdon, Mariella Zunker, Jan Niclas Wolf, Kai Prüfer, Jörg Ackermann, Christoph Welsch, Ina Koch

**Affiliations:** Goethe University Frankfurt, Molecular Bioinformatics, Institute of Computer Science, Faculty of Computer Science and Mathematics, 60325 Frankfurt am Main, Germany; Goethe University Frankfurt, Molecular Bioinformatics, Institute of Computer Science, Faculty of Computer Science and Mathematics, 60325 Frankfurt am Main, Germany; Goethe University Frankfurt, Molecular Bioinformatics, Institute of Computer Science, Faculty of Computer Science and Mathematics, 60325 Frankfurt am Main, Germany; Goethe University Frankfurt, Molecular Bioinformatics, Institute of Computer Science, Faculty of Computer Science and Mathematics, 60325 Frankfurt am Main, Germany; Goethe University Frankfurt, Molecular Bioinformatics, Institute of Computer Science, Faculty of Computer Science and Mathematics, 60325 Frankfurt am Main, Germany; Goethe University Frankfurt, University Hospital, Medical Clinic 1, 60590 Frankfurt am Main, Germany; Goethe University Frankfurt, Molecular Bioinformatics, Institute of Computer Science, Faculty of Computer Science and Mathematics, 60325 Frankfurt am Main, Germany

## Abstract

**Motivation:**

The functional complexity of biochemical processes is strongly related to the interplay of proteins and their assembly into protein complexes. In recent years, the discovery and characterization of protein complexes have substantially progressed through advances in cryo-electron microscopy, proteomics, and computational structure prediction. This development results in a strong need for computational approaches to analyse the data of large protein complexes for structural and functional characterization. Here, we aim to provide a suitable approach, which processes the growing number of large protein complexes, to obtain biologically meaningful information on the hierarchical organization of the structures of protein complexes.

**Results:**

We modelled the quaternary structure of protein complexes as undirected, labelled graphs called complex graphs. In complex graphs, the vertices represent protein chains and the edges spatial chain–chain contacts. We hypothesized that clusters based on the complex graph correspond to functional biological modules. To compute the clusters, we applied the Leiden clustering algorithm. To evaluate our approach, we chose the human respiratory complex I, which has been extensively investigated and exhibits a known biological module structure experimentally validated. Additionally, we characterized a eukaryotic group II chaperonin TRiC/CCT and the head of the bacteriophage Φ29. The analysis of the protein complexes correlated with experimental findings and indicated known functional, biological modules. Using our approach enables not only to predict functional biological modules in large protein complexes with characteristic features but also to investigate the flexibility of specific regions and coformational changes. The predicted modules can aid in the planning and analysis of experiments.

**Availability and implementation:**

Jupyter notebooks to reproduce the examples are available on our public GitHub repository: https://github.com/MolBIFFM/PTGLtools/tree/main/PTGLmodulePrediction.

## 1 Introduction

The structural and functional complexity of biological processes is strongly related to the assembly of proteins into protein complexes, such as the human nuclear pore complex, or complexes of the respiratory chain. The discovery and structural characterization of protein complexes in recent years have substantially progressed through advances in cryo-electron microscopy (Chua *et al.* 2022, Guaita *et al.* 2022) and proteomics (Low *et al.* 2021). Last year alone, the Protein Data Bank (Berman *et al.* 2000) released almost 1300 entries with more than ten chains, i.e. about 13% of the total number of entries released in that year. Additionally, the enhanced computational modelling of protein structures using deep learning methods (Baek *et al.* 2021; Jumper *et al.* 2021; Lin *et al.* 2023) has accelerated the computational prediction of protein complexes and the development of combined computational and experimental approaches (Evans *et al.* 2021, Humphreys *et al.* 2021, Bryant *et al.* 2022, Drake *et al.* 2022, Fontana *et al.* 2022, O‘Reilly *et al.* 2023). It is expected that the number of protein complex structures will rapidly grow. Since the analysis of protein complexes can be challenging, it is essential to develop computational tools to process a large number of data and extract relevant information for structural and functional characterization.

We have developed the Protein Topology Graph Library (PTGL) (Wolf *et al.* 2020), a library with tools to model protein structures as undirected, labelled graphs on various levels of abstraction. The graphs represent the protein structure on the level of amino acids, secondary structure elements, or protein chains. We focus on the level of protein chains represented by the complex graph. In the complex graph, the vertices represent protein chains and the edges spatial contacts between chains. The edges are labelled by the number of contacts. In this work, we hypothesized that partitioning of the protein complex graph by graph-theoretical methods gives functional, biological modules. To show the prediction of biologically meaningful modules using our method, we analysed three protein complexes with sizes of 16, 45, and 400 chains. We were able to describe characteristic structural features, flexible regions, and conformational changes. We developed Jupyter Notebooks, which applied the analysis to three exemplary complexes and could be adapted to analyse other protein complexes.

## 2 Materials and methods

We modelled protein structures as complex graphs based on the quaternary structure of protein complexes, using the PTGL (Wolf *et al.* 2020), and applied graph-theoretical clustering methods. The detailed workflow is provided in Jupyter Notebooks (Granger and Pérez 2021) on our public GitHub repository (https://github.com/MolBIFFM/PTGLtools/tree/main/PTGLmodulePrediction).

### 2.1 Preparation of protein structures

We considered the PDB (Berman *et al.* 2000) entries of the human respiratory complex I, PDB ID 5XTD (Guo *et al.* 2017), the group II chaperonin TRiC/CCT in an open conformation, PDB ID 7YLV (Han *et al.* 2023), and closed conformation, PDB ID 7YLX (Han *et al.* 2023), and the head of the bacteriophage Φ29, PDB ID 6QYD (Xu *et al.* 2019), all in the macromolecular Crystallographic Information Format. The structures were available in the biological assembly and had a resolution below 4.0 Å. Secondary structure assignments, as required for the PTGL, were assigned according to the DSSP algorithm (Kabsch and Sander 1983, Touw *et al.* 2014) (https://github.com/PDB-REDO/dssp, version 4.4).

### 2.2 Complex graphs

We define a protein complex graph, G=(V,E,W), as undirected, labelled graph with *V* as the set of vertices, representing the chains of the protein, *E* as the set of edges, indicating spatial residue-residue contacts between the chains, and *W* as edge weights for the number of residue-residue contacts between the considered chains, according to Wolf *et al.* (2020). We used a sphere model for atoms, with a radius of 2.0 Å for protein atoms and 3.0 Å for ligand atoms; see our previous publication (Wolf *et al.* 2020). Two atoms were in contact if their spheres overlap. Two amino acid residues were in contact if they share at least one atom-atom contact. Two protein chains were in contact if they share at least one residue-residue contact. We used the tools of PTGL (https://github.com/MolBIFFM/PTGLtools) to compute complex graphs and stored the graphs in the Graph Modelling Language format.

### 2.3 Computation of structural modules

A clustering of the complex graph, G=(V,E,W), is given by a partition $M=\{M_{1},\ldots ,M_{k}\}$ with $M_{i}\subseteq V,\;i=1,\ldots ,k$, and *k* as the number of clusters. For the partitioning of the complex graphs, we applied the Leiden algorithm (Traag *et al.* 2019) in combination with the Q-modularity optimization (Newman and Girvan 2004, Reichardt and Bornholdt 2006). The Leiden algorithm considers an undirected graph and is a modification of the Louvain algorithm (Blondel *et al.* 2008). The Leiden algorithm is an agglomerative procedure and starts with a singleton partition of the graph with each vertex forming its own cluster. The Leiden algorithm consists of three iterative steps: local movement, refinement, and aggregation. In the local movement phase, a node may be moved to any cluster for which the Q-modularity increases. The cluster, to which a vertex is moved, is selected randomly and the larger the increase in the Q-modularity, the more likely a cluster is selected. In the refinement phase, each cluster may be split into multiple clusters. In the aggregation phase, a new graph is constructed with each vertex representing a cluster of the refined partition. The new graph is partitioned based on the nonrefined partition and is used as input for the local movement phase. The steps are iterated until no further improvement can be obtained.

The complex graphs were processed with the iGraph package (Csardi and Nepusz 2006). The implementation of the Leiden algorithm applies the generalized modularity (Reichardt and Bornholdt 2006) with an adjustable resolution parameter. We set the resolution parameter to 1.0 and the number of iterations to −1 to run until convergence was reached. Based on initial numerical tests, we performed 2000 independent computations for each complex graph, since the order in which the vertices in the implementation are processed is random, which could lead to variations in the results. We chose the partition, *M*, with the highest value of Q-modularity and called the clusters, $M_{1},\ldots ,M_{k}$, structural modules.

### 2.4 Data visualization

The visualization of the complex graphs was performed with the iGraph library (Csardi and Nepusz 2006). Protein structures were visualized using PyMOL (Schrödinger, LLC 2022).

## 3 Results and discussion

### 3.1 Human respiratory complex I

The human respiratory complex I (rCI) is the first protein complex of the respiratory chain and is essential to form a proton gradient across the membrane. The human rCI is an extensively investigated protein complex with 44 unique protein chains and 45 chains in total. Human rCI was chosen as the first case study to evaluate our approach since functional, biological modules have been assigned and discussed in several experimental studies (Brandt 2006, Hunte *et al.* 2010, Stroud *et al.* 2016, Guerrero-Castillo *et al.* 2017).

**Figure 1.**
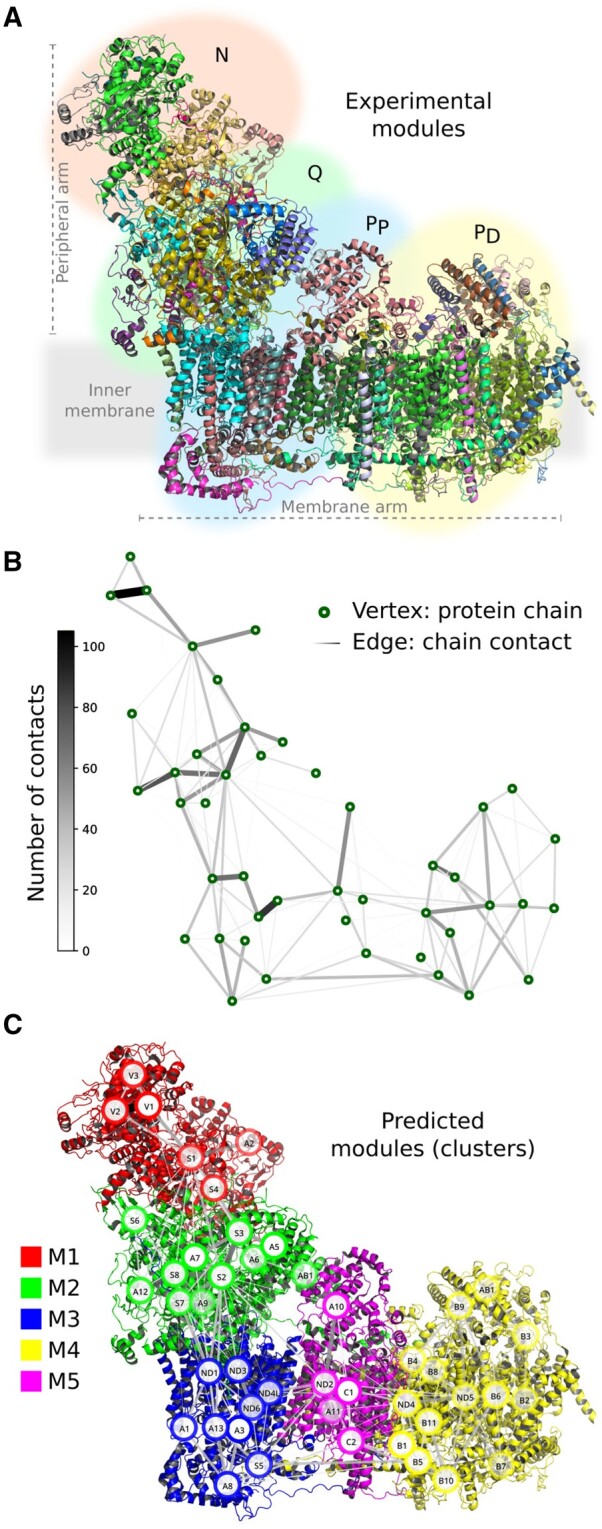
Graph-theoretical analysis of the human respiratory complex I (rCI, PDB ID 5XTD). (A) Experimentally assigned biological modules of rCI, the N-module, the Q-module, the proximal P${\;}_{P}$-module, and the distal P${\;}_{D}$-module. (B) Protein complex graph with individual chains represented as vertices and placed at the respective geometrical centre. Chain–chain contacts are represented as edges between vertices. (C) Structural modules assigned to the quaternary structure of rCI superimposed by the underlying graph structure. The abbreviated gene names of the respective protein chains are used as vertex names (see Supplementary Table S2).

Figure 1A shows the known module structure of rCI. The peripheral arm consists of the N-module and the Q-module. In the N-module, oxidation of NADH takes place under the release of two electrons. In the Q-module, ubiquinone is bound onto which the electrons are transferred (Brandt 2006). Several models are discussed for the coupling mechanism between the electron transfer in the peripheral arm and the translocation of protons across the membrane. The membrane arm, or P-module, is grouped into a proximal and a distal part, P${\;}_{P}$ and P${\;}_{D}$, respectively. The proton translocation across the membrane involves three antiporter-like subunits. Two of the three antiporter-like subunits, ND4 and ND5, are located in the P${\;}_{D}$-module, and the third antiporter-like subunit, ND2, is located in the P${\;}_{P}$-module (Hunte *et al.* 2010).

Figure 1B shows the complex graph of the quaternary structure of rCI. The number of contacts between the protein chains is represented by the thickness and the colour of the edges. Well-connected regions in the graph can be observed and approximate experimentally assigned biological modules.

Figure 1C shows the obtained structural modules, M1 to M5, which are mapped onto the quaternary structure of rCI; see Supplementary Table S2 for details. The structural modules are in accordance with the well-connected regions in the complex graph and correlated with experimental findings.

#### 3.1.1 Peripheral arm

The structural modules M1 and M2 in Fig. 1C represent the peripheral arm of rCI and correspond to the biological N-module and Q-module, respectively. The accessory subunits S6 and A12 are associated with the N-module and are involved in the final step of the assembly (Stroud *et al.* 2016, Guerrero-Castillo *et al.* 2017). The extensive interaction of S6 and A12 has implied that these subunits operate as one unit, which ensures the release of an assembly factor after the N-module binds (Parey *et al.* 2019). Structurally, the subunits S6 and A12 are located at the interface between the N-module and the Q-module; see a highlighted representation in Supplementary Fig. S1. The connections of S6 and A12 with the Q-module are more pronounced compared to the connection with the N-module, especially the contact of A12 with the subunit S8. Our approach assigns S6 and A12 to the Q-module. The functional, biological structure of the peripheral arm of rCI is well-represented by the structural modules of our approach.

#### 3.1.2 Membrane arm

The structural modules M3, M4, and M5 in Fig. 1C represent the membrane arm of rCI. The module M4 exactly corresponds to the P${\;}_{D}$-module, which contains the two antiporter-like central subunits ND4 and ND5. The modules M3 and M5 together represent the P${\;}_{P}$-module. Based on the analysis of the assembly pathway, the P${\;}_{P}$-module has been further divided into the submodules P${\;}_{P}$-a and P${\;}_{P}$-b (Guerrero-Castillo *et al.* 2017). The module M3 resembles P${\;}_{P}$-a, which harbours the central subunit ND1. The module M5 resembles P${\;}_{P}$-b, which contains the central subunit ND2. The smaller subunits ND3, ND4L, and ND6 are located between the central subunits ND1 and ND2 and have been associated with the biological submodule P${\;}_{P}$-b since they assemble with ND2, C1, and C2. With our approach, the subunits ND3, ND4L, and ND6 are associated with the module M3, which corresponds to P${\;}_{P}$-a.

The biological modules for complex I have been assigned according to evolutionary relationships. Additionally, functional modules in protein complexes might extend beyond the interface of the evolutionarily evolved modules during complex assembly. The cellular organization can be described by a hierarchy of functional modules (Pereira-Leal *et al.* 2006). On the level of the protein interaction network, the functional modules are protein complexes. The proteins within stable complexes show more and/or stronger connections compared to other proteins in the network. The same principle can be applied to the functional modules within large protein complexes, which have assembled from evolutionarily evolved smaller protein complexes. Regarding functional modules in protein interaction networks, it has been concluded that a functional module is not necessarily an evolutionary module (Snel and Huynen 2004, Pereira-Leal *et al.* 2006). Thus, the functional modules within protein complexes might be formed by the well-connected subunits within the complete protein complex.

There is an ongoing discussion in the literature about a fourth proton translocation pathway, which might not be associated with evolutionarily evolved modules. Besides the three antiporter-like subunits involved in the proton translocation, a fourth translocation pathway has been assumed to be either at the interface of subunits ND3, ND4L, and ND6, or to involve the substructure referred to as E-channel (Baradaran *et al.* 2013, Kampjut and Sazanov 2020, Parey *et al.* 2021). The entrance of the E-channel has been located at the interface of the subunits S2 of the Q-module and ND1 of the P${\;}_{P}$-module and has been shown to extend toward the interface with subunit ND6, establishing a connection involving the subunits ND1, ND3, ND4L, and ND6 (see Supplementary Fig. S2). In Fig. 1B, pronounced interactions between the respective subunits are represented by high edge weights. The structural module M3 is larger compared to the experimentally assigned module P${\;}_{P}$-a. Based on the assumption that biological modules are internally well connected, the structural module M3 could represent a functional unit for proton translocation.

The structural modules represented the general biological partition of the membrane arm. The differences in the region where a fourth proton translocation has been assumed might indicate a modular structure with a biological function.

#### 3.1.3 Identification of flexible regions

Regions with higher edge weights between subunits were well-connected and could indicate comparably rigid parts of the protein complex. We assumed that the structural modules were rather rigid. Between the modules, we expected higher flexibility. The biological P${\;}_{P}$-module, for instance, was clustered into two modules, M3 and M5, which might indicate a higher flexibility at the interface. Flexibility between M3 and M5 might predict conformational movements similar to those of the open and closed forms of rCI, which have been shown to occur at the interface of the subunits ND1 and ND6 (Kampjut and Sazanov 2020).

The simulation of the flexibility of large protein complexes with methods such as molecular dynamics is time-consuming. Protein flexibility can be predicted using computational structure prediction methods such as AlphaFold (Jumper *et al.* 2021, Ma *et al.* 2023), but the methods are limited when protein complexes are considered. Additionally, the more flexible a monomeric protein is, the larger the conformational change could be upon binding in a protein complex (Marsh *et al.* 2012). The monomer flexibility might no longer be present within the protein complex. Our method provides a fast estimate of flexible regions within protein complexes. A more detailed experimental or computational analysis can focus on the estimated regions.

The first case study showed that structural modules were in good agreement with experimental findings. We quantitatively evaluated the agreement using the adjusted Rand index (Hubert and Arabie 1985). We restricted the quantitative analysis to the first case study since a complete and consistent experimental reference for the modular structure of a protein complex is required. For complex I, there is an experimentally determined consensus about functional modules, except for the fourth proton translocation pathway, leading to a limitation of the quantitative comparison.

We compared the structural modules with the above-described experimental reference without (1) and with (2) submodules: (1) modules N, Q, P${\;}_{P}$, and P${\;}_{D}$ and (2) modules N, Q, P${\;}_{P}-a$, P${\;}_{P}-b$, P${\;}_{D}-a$, and P${\;}_{D}-b$. Respectively, the adjusted Rand indices were 0.79 and 0.60; see the modular structure for references (1) and (2) in Supplementary Table S2. The agreement with the experimental reference (2) considering submodules was moderate. The peripheral arm of complex I was well-represented, but the structural modules in the membrane arm did not represent the submodules P${\;}_{D}-a$ and P${\;}_{D}-b$, and partly the submodules P${\;}_{P}-a$ and P${\;}_{P}-b$. Functional modules might not completely resemble modules based on evolutionary relationships; see above. The agreement with the experimental reference (1), considering no submodules, was good. The modular structure of the membrane arm was resembled, except for a region, which is still under discussion. The structural modules for this region might be biologically meaningful; see above.

### 3.2 Group II chaperonin TRiC/CCT

The group II chaperonin TCP-1 ring complex/chaperonin containing TCP-1 (TRiC/CCT) from yeast with co-chaperon plp2 is a comparatively small protein complex with 17 chains, which is commonly associated with the folding of the cytoskeletal proteins actin and tubulin. TRiC/CCT was chosen for the second case study since it is highly asymmetrical despite the pseudosymmetrical appearance, which makes it special among other chaperones (Gestaut *et al.* 2019). Additionally, TRiC/CCT has a unique ATP-driven cycle, which involves conformational movements. ATP binding leads to a compact open state and ATP hydrolysis effects the closure of the chamber and encloses the substrate. Thereby, the subunits of one ring have shown positive cooperativity in ATP binding and have been coupled as one functional unit (Horovitz *et al.* 2001, Kafri *et al.* 2001).

TRiC/CCT from yeast has been structurally resolved in different states of the folding process (Han *et al.* 2023). The complex consists of nine unique protein chains. The chaperonin chamber is formed by two identical rings each composed of eight paralog chains. Additionally, the co-chaperone plp2 is located inside the chamber.

We analysed two quaternary structures, an open and a closed conformation, each containing the co-chaperon plp2 and the substrate tubulin or actin. The structure of the substrates has not been resolved, but the electron density indicates their positions. Thus, the quaternary structure of TRiC/CCT was in the appropriate state in the folding process, but the complex graph was not biased by the different folding states of the substrate.

**Figure 2.**
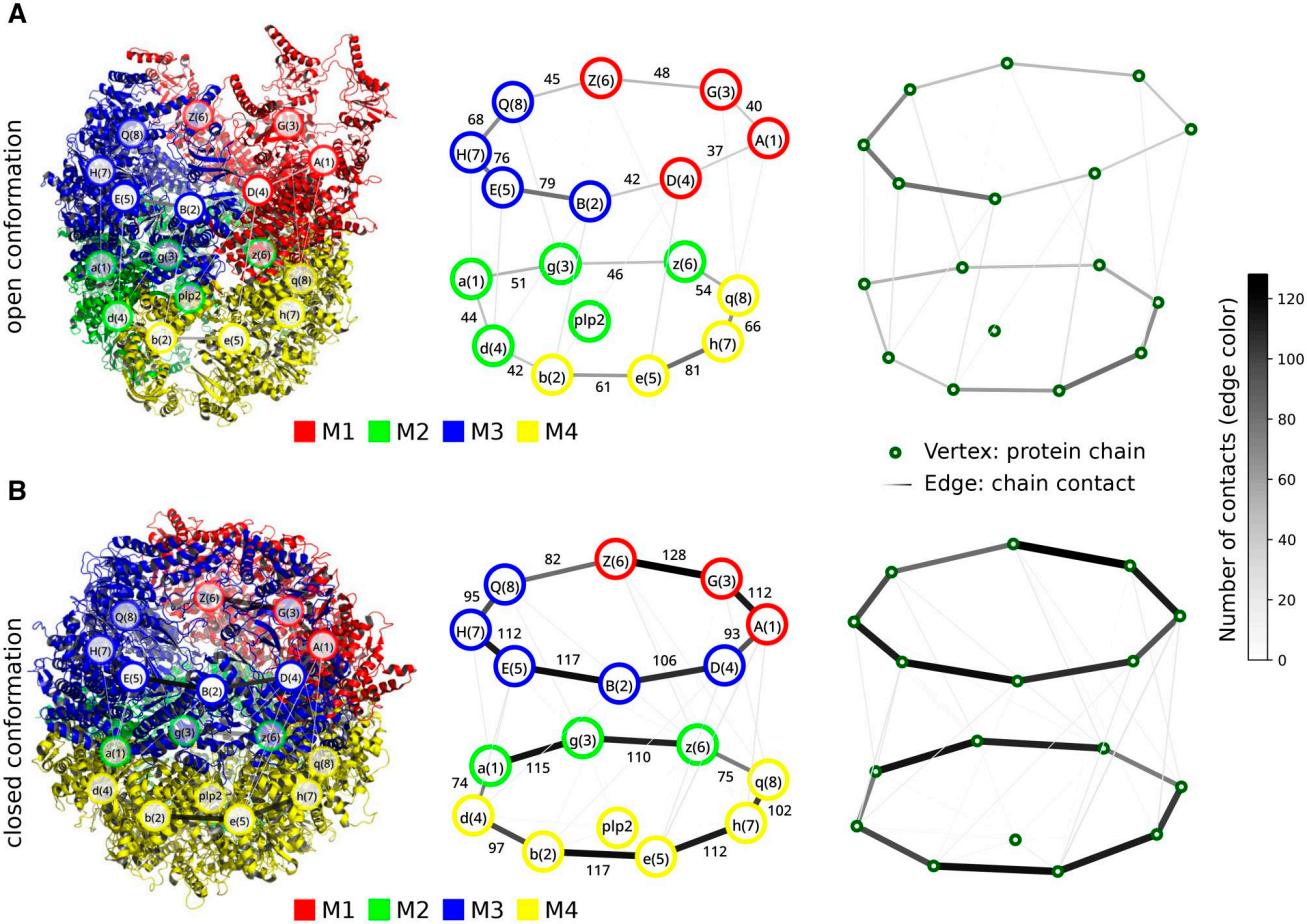
Graph-theoretical analysis of the group II chaperonin TCP-1 ring complex/Chaperonin containing TCP-1 (TRiC/CCT) from yeast with co-chaperon plp2 in an open (A, PDB ID 7YLV) and a closed conformation (B, PDB ID 7YLX). *Left*: Structural modules assigned to the quaternary structure and superimposed by the underlying graph structure. *Centre* and *right*: Protein complex graph with individual chains represented as vertices and placed at the respective geometrical centre. Chain–chain contacts are represented as edges between vertices. Numbers at edges in the centre figure are the computed chain–chain contacts above a minimum number of 30 contacts.

The right parts of Fig. 2A and 2B illustrate the complex graphs for the open and closed conformations, respectively. In correlation with the experimentally assigned functional units, the eight subunits within each ring are well-connected in the graph structure; see pictures in the centre and on the right. The intra-ring contacts between the subunits are pronounced in comparison to the inter-ring contacts, especially in the closed conformation. Further, the conformations show a certain asymmetry in the number of contacts between the subunits within a ring.

The left and centre pictures of Fig. 2 depict the results of the cluster analysis. For each conformation, four structural modules are predicted. The modular structure changes from the open to the closed conformation; see Supplementary Tables S3 and S4 for details. The obtained structural modules separate the two rings from each other, but each ring is further partitioned into two modules. The partitioning in both rings of each conformation is identical, even though the co-chaperone plp2 is not centred within the complex.

The co-chaperone plp2 and the substrate occupy opposite rings of the complex (Han *et al.* 2023). In the open conformation, plp2 is interacting with the subunits 3, 1, and 4 of its ring. Accompanying closure of the complex from the open to the closed conformation of TRiC/CCT, plp2 is translocating to the subunits 4, 2, 5, and 7 of its ring. The centre pictures of Fig. 2 show plp2, occupying the same ring in each conformation. In the open conformation, plp2 is assigned to module M2 with subunits 6, 3, 1, and 4; see the centre picture of Fig. 2A. In the closed conformation, plp2 is assigned to module M4 with subunits 4, 2, 5, 7, and 8; see the centre picture of Fig. 2B. The translocation of plp2 is depicted in the predicted modular structure.

From the results in Fig. 2, we propose two structural modules in each ring and a dynamic change in the module structure upon chamber closure. Experimentally, each ring of the chamber has been further separated according to the distribution of conserved surface charges. A negative and a positive hemisphere have been assigned to the subunits 5, 2, and 4, and subunits 3, 6, and 8, respectively (Leitner *et al.* 2012). The positive hemisphere has also been extended by subunit 1 (Gestaut *et al.* 2019, Han *et al.* 2023). Additionally, the subunits 3, 6, and 8 exhibiting low ATP binding and hydrolysis rates have been associated to bind the substrate, while the other hemisphere is related to ATP hydrolysis (Leitner *et al.* 2012, Reissmann *et al.* 2012, Kalisman *et al.* 2013). Since the experimental findings concern the folding process of the substrate, we compared them with the structural modules in the closed conformation, where the main folding process takes place, and focused the analysis on the ring, which contains the substrate.

Module M1 in the centre picture of Fig. 2B in the closed conformation clusters the subunits 1, 3, and 6. The result correlates with the experimentally assigned positive hemisphere except for subunit 8. The number of contacts between the neighbouring subunits 6 and 8 is clearly smaller compared to the contacts between the subunits 1, 3, and 6. The smaller number of contacts explains the partitioning results. An interpretation of the results may be higher flexibility, which might be required between the subunits 6 and 8. Nevertheless, our approach partitions the protein complex comparably to experimental findings and highlights a potentially interesting region.

A comparison of the centre pictures in Fig. 2 shows that subunit 4 changed its assignment from module M1 to module M3 from the open to the closed conformation, respectively. As the only subunit, subunit 4 exhibits an outward-pointing N-terminus, which is located between the subunits 4 and 2 and has been described as a conserved structural feature (Leitner *et al.* 2012). Moreover, subunit 4 has been considered to be a key to regulating the ATP-driven cycle (Reissmann *et al.* 2012) and might be part of a different biological module upon the closure of the chamber, as observed in our analysis.

The second case study illustrated the analysis of a protein complex in different conformations. We predicted an asymmetrical module structure, which was comparable to experimental findings. We located a key subunit in the complex and showed the sensitivity of our approach according to conformational changes in the protein complex. The analysis could be extended to trajectories of molecular dynamics simulations. With an analysis of the dynamic behaviour in protein complexes, interesting regions could be identified for experimental studies.

### 3.3 Bacteriophage Φ29

The head of the mature bacteriophage Φ29 is a very large protein complex with 400 chains (Xu *et al.* 2019). The structure consists of only two unique protein chains, gp8 and gp8.5. The major capsid protein, gp8, assembles into pentameric and hexameric capsomers and the minor capsid protein, gp8.5, into trimeric head fibres. The fibres exhibit a pseudohexagonal base and interact with one pentameric capsomer and two hexameric capsomers. The head of Φ29 was chosen as the third case study since it was a very large protein complex with little variation in the individual components, which assemble into different structural units.

**Figure 3.**
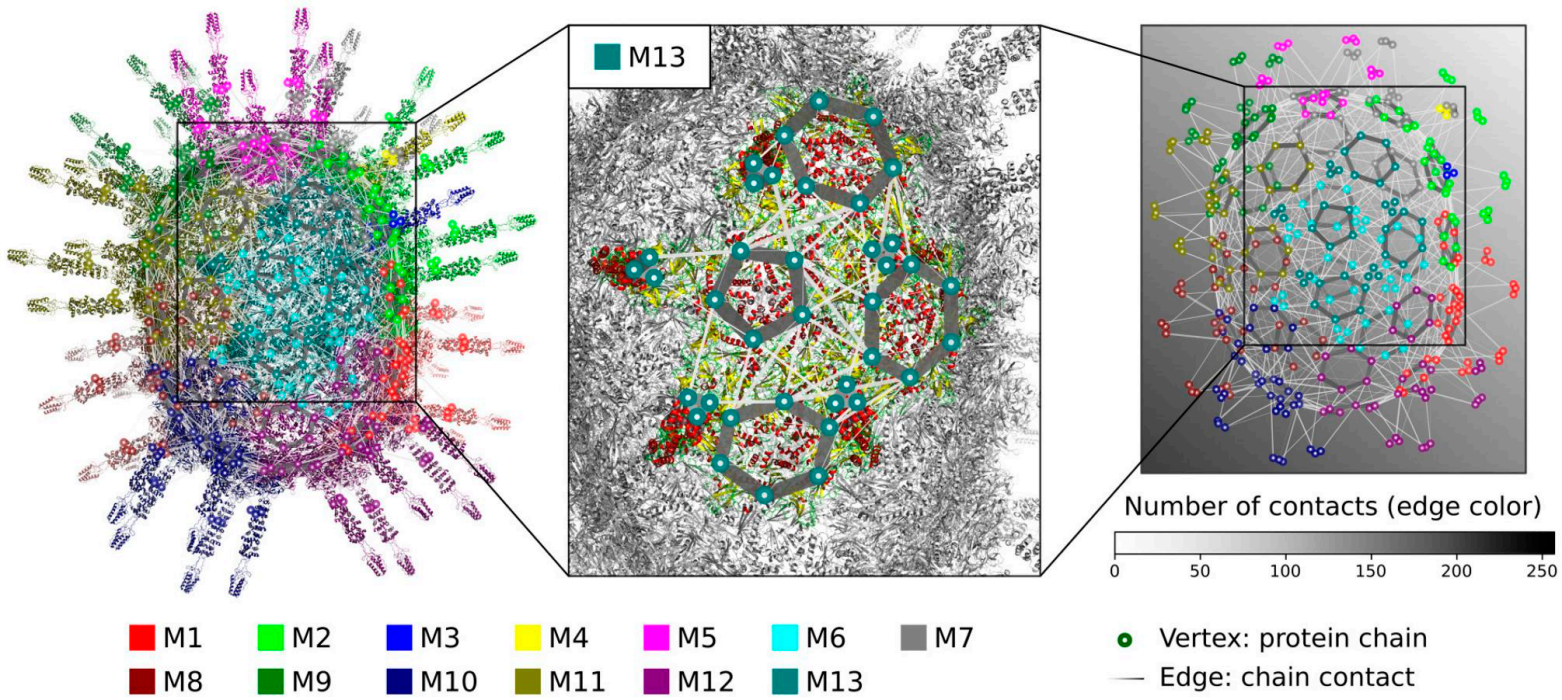
Graph-theoretical analysis of the head of the bacteriophage Φ29 (PDB ID 6QYD). *Left*: Structural modules assigned to the quaternary structure and superimposed by the underlying graph structure. *Right*: Protein complex graph with individual chains represented as vertices and placed at the respective geometrical centre. Chain–chain contacts are represented as edges between vertices. *Centre*: Detailed view of the structural module M13 as overlay of the respective part of the graph and the highlighted cartoon structure (red: helices; yellow: strands; green: coils).

The complex graph in Fig. 3 depicts the arrangement of the capsid proteins into trimeric, pentameric, and hexameric structures. The regular arrangements are in agreement with experimental results. The computed structural modules include the regular arrangements and could be interpreted as a higher-level modular structure. The computed modules include one pentameric capsomer surrounded by five trimeric fibres and flanked on one side by three hexameric capsomers. The modular structure is correlated with about one-half of the asymmetric unit, which has been determined in the crystal structure. Since the asymmetric unit represents a repetitive part of the protein crystal, presumably without major variations, the correlation supported the structural module to represent a certain structural unit that might be interpreted as a pattern. Further, we can observe variations of the modular structure as seen for the structural modules M3 and M4, which are two trimeric fibres, each assigned to an individual module. The result emphasizes a region, which might be connected in a different way, maybe motivating future studies.

The third case study demonstrated that very large structures could be analysed with our approach, and the underlying structure of a highly repetitive assembly could be observed in the complex graph. The clustering predicted a higher-level modular structure and emphasized regions with variations, which could be relevant for experimental analysis or for applications at the nanoscale (Paczesny and Bielec 2020), where features of the capsid are of great relevance.

### 3.4 Execution time

The prediction of biological modules with our method required two main steps, the computation of the complex graph from the quaternary structure of a protein complex and the partitioning of the complex graph. The graph partitioning was performed in less than two seconds for each case study. The time-consuming step was the computation of the complex graph. The complex graphs for complex I and TRiC/CCT were computed in about 20 s. The computation of a large number of complex graphs of similar sizes is feasible. For the very large protein structure of the bacteriophage Φ29 with 400 protein chains, the complex graph was computed in about 1.5 h.

All calculations for this paper were performed on a desktop PC with an Intel Xeon CPU E3-1246 v3 and 16.0 GB RAM using a single core. The detailed execution time for each case study was described in the Supporting Information, Supplementary Table S1.

### 3.5 Computational limitation of Q-modularity optimization

For the computation of structural modules within protein complexes, we applied the Leiden algorithm using Q-modularity optimization (Newman and Girvan 2004, Reichardt and Bornholdt 2006, Traag *et al.* 2019). Clusters obtained by the Leiden algorithm are internally well-connected, which is the assumption for biological modules in our hypothesis. The Leiden algorithm has been applied successfully to characterize structural features in biological fields, spanning from the clustering of complete cells into groups for cell cycle analysis (Stallaert *et al.* 2022) to the partitioning of small molecules to estimate molecular properties (Martinez-Hernandez *et al.* 2021).

However, it is well-known that modularity optimization suffers a resolution limit (Fortunato and Barthélemy 2007, Lancichinetti and Fortunato 2011). Depending on the size of the graph, computed clusters might contain smaller clusters or might be a combination of more than one cluster. As a consequence, it is not clear whether a structural module in the complex graph further consists of smaller modules or is partitioned into too many modules. The generalized modularity (Reichardt and Bornholdt 2006) partially approaches the problem of the resolution limit. Developments in the field of community detection are still ongoing (Dey *et al.* 2021). Although the structural modules might show differences compared to experimental results, we obtained biologically reasonable partitions for the case studies.

## 4 Conclusion

We modelled protein complexes as undirected, labelled graphs called complex graphs. We introduced an approach, which applies graph-theoretical clustering methods to protein complex graphs to predict biological, functional modules. The results correlated well with experimental findings, which might make our approach suitable to characterize unknown protein complexes. The presented method is fast and can be applied on a larger scale.

We conclude that obtained partitions from our approach give information about functional, biological modules, and central structural arrangements. Moreover, well-connected regions in the complex graph correlated with more rigid parts and allowed the investigation of flexible regions. The results demonstrated that a graph-theoretical prediction of biological modules is meaningful. Future studies might also apply the comparison of structural modules for the classification of protein complexes or detection of functional motives of protein complexes among different organisms. The graph-theoretical analysis of protein complexes facilitates a wide range of applications.

## Supplementary Material

btae112_Supplementary_Data

## Data Availability

The data availability statement is included at the end of the abstract.
