# Supplementary material for: Graph-theoretical prediction of biological modules in quaternary structures of large protein complexes
Source: Bioinformatics. 2024 Mar 6;40(3):btae112. doi: 10.1093/bioinformatics/btae112 (PMC11212496; doi:10.1093/bioinformatics/btae112)
Supplement: btae112_Supplementary_Data [file btae112_supplementary_data.pdf]

Supporting Information

Graph-theoretical prediction of biological modules in quaternary structures of large protein complexes

Florian J. Gisdon[1,*] **Mariella Zunker,**[1] **Jan Niclas Wolf,**[1] **Kai Prüfer,**[1] **Jörg Ackermann,**[1] **Christoph Welsch**[2] **and Ina Koch**[1,*]

[1]Molecular Bioinformatics, Institute of Computer Science, Faculty of Computer Science and Mathematics, Goethe University Frankfurt, 60325 Frankfurt am Main, Germany and [2]Department of Internal Medicine 1, University Hospital Frankfurt, Goethe University Frankfurt, 60325 Frankfurt am Main, Germany

*Corresponding author.
Contact: florian.gisdon@bioinformatik.uni-frankfurt.de or ina.koch@bioinformatik.uni-frankfurt.de

1. Supporting figures

Figure S1 shows the quaternary structure of the human respiratory complex I, PDB ID 5XTD (Guo et al., 2017). Structural modules were computed as described in the original paper. The structural modules M1 to M5 are mapped onto the protein complex structure, which is coloured accordingly. The subunits S6 and A12 are assigned to the structural module M2, see Table S2, and are highlighted in the figure in cyan and in orange, respectively.

Figure S2 shows the structure of human respiratory complex I with the protein chains of a well-connected part highlighted in green and blue, according to the structural modules in Figure S1. The structure is overlayed by the subgraph of the complex graph for the highlighted protein chains. The vertices of the subgraph are labelled with the abbreviated gene names of the corresponding protein chains, see Table S2. The edges are coloured orange. The thickness of the edges represents the number of contacts, see Figure 1 in the original paper. The subgraph is well-connected and correlates with the position of a functional part in the protein complex, the E-channel. The approximate position of the E-channel is indicated as the magenta region.

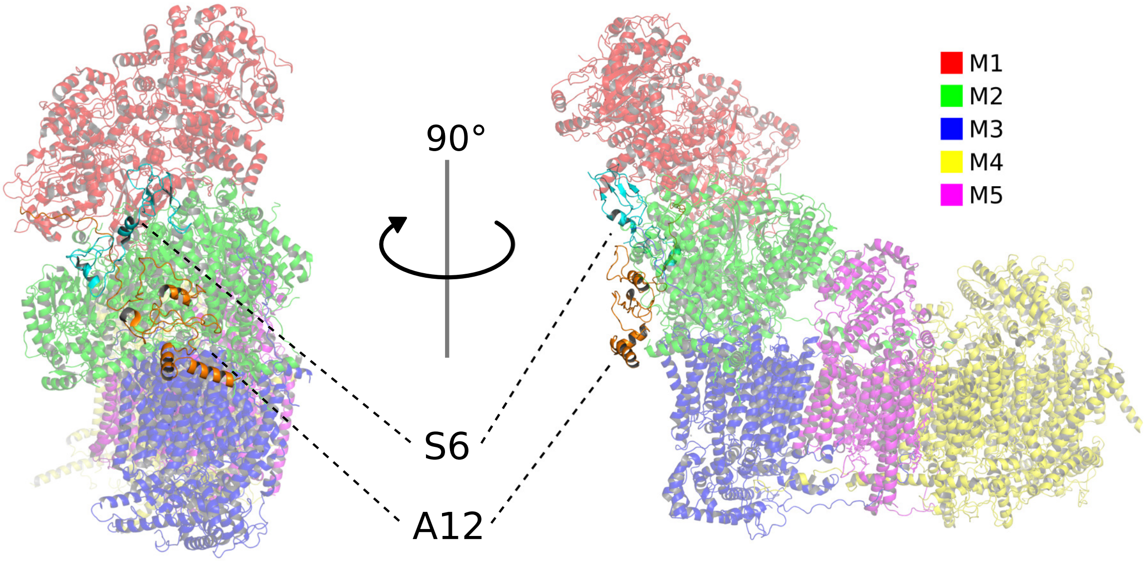

Fig. S1: Protein complex structure of the human respiratory complex I, PDB ID 5XTD (Guo et al., 2017), coloured according to the structural modules M1 to M5. The subunits S6 and A12 are highlighted to show the position in the protein complex.

© The Author . Published by Oxford University Press. All rights reserved. For permissions, please e-mail: journals.permissions@oup.com

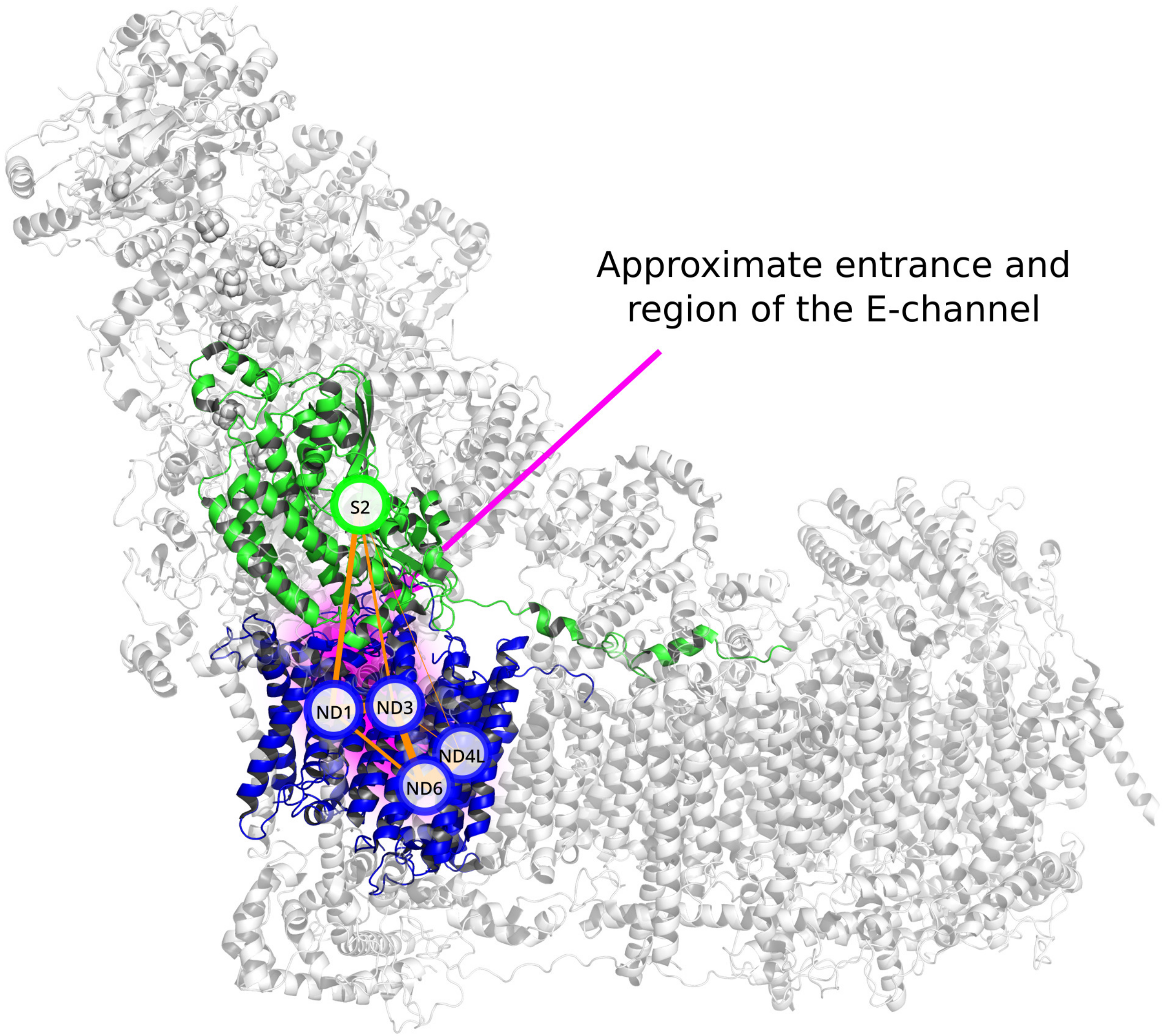

Fig. S2: Protein complex structure of the human respiratory complex I, PDB ID 5XTD (Guo et al., 2017). The highlighted protein chains are overlayed by a subgraph of the complex graph. The subgraph is well-connected and correlates with the position of the E-channel in the protein structure. The colours of the highlighted protein chains correspond to the structural modules, see Figure 1 in the original paper. The approximate position of the E-channel is indicated as the magenta region. The edges of the subgraph are colored in orange. The thickness of the edges represents the number of contacts, see Figure 1 in the original paper.

2. Supporting tables

2.1. Execution time

The prediction of biological modules based on complex graphs consists of two main steps, the computation of the complex graph from the quaternary structure of the protein complex, and the partitioning of the complex graph. In Table S1, we provide the execution time for the complex graph computation, the graph partitioning with the Leiden algorithm, and the total runtime for the part of the module prediction in the Jupyter Notebooks. The execution time for individual cells of the Jupyter Notebooks is provided within each cell as comments. The execution time for the interactive analysis is not provided in Table S1.

Table S1. The execution time for the module prediction measured on a desktop PC with Intel(R) Xeon(R) CPU E3-1246 v3 @ 3.50GHz and 16.0 GB RAM.

Protein complex	Complex graph computation [s]	Graph partitioning [s]	Total execution time [s]
Complex I (PDB ID 5XTD)	24.30	0.46	31.55
Chaperonin TRiC/CCT (PDB ID 7YLV) open conformation	16.09	0.16	19.67
Chaperonin TRiC/CCT (PDB ID 7YLX) closed conformation	19.56	0.16	23.51
Bacteriophage Φ29 (PDB ID 6QYD)	5304.13	1.95	5893.52

2.2. Affiliation of protein chains with computed structural modules

The tables S2 to S5 list the protein chains of the respective protein complexes and show the association with the structural modules. For the human respiratory complex I in Table S2, the gene names and the abbreviated gene names are provided in addition. Biological modules for complex I have been assigned and discussed in several experimental studies (Brandt, 2006; Hunte et al., 2010; Stroud et al., 2016; Guerrero-Castillo et al., 2017). We compared the structural modules with the experimental reference without (1) and with (2) submodules: (1) modules N, Q, $\mathrm{P_P}$, and $\mathrm{P_D}$, and (2) modules N, Q, $\mathrm{P_P} - a$, $\mathrm{P_P} - b$, $\mathrm{P_D} - a$, and $\mathrm{P_D} - b$. For the protein complexes of the group II chaperonin TRiC/CCT, Table S3 and Table S4, a common numbering of the protein chains is provided in addition.

Table S2. Protein chain IDs and their associated gene names of the human respiratory complex I, PDB ID 5XTD (Guo et al., 2017), and the respective affiliation with structural modules, see Figure 1 in the original paper. We compared the structural modules with the experimental reference without (1) and with (2) submodules: (1) modules N, Q, P_P, and P_D, and (2) modules N, Q, $P_P - a$, $P_P - b$, $P_D - a$, and $P_D - b$, see assignment and discussion in Brandt (2006); Hunte et al. (2010); Stroud et al. (2016); Guerrero-Castillo et al. (2017).

Chain name	Gene name	Abbreviated gene name	Structural module	Reference (1)	Reference (2)
A	NDUFV1	V1		N	N
F	NDUFA2	A2		N	N
K	NDUFV3	V3	M1	N	N
L	NDUFS4	S4		N	N
M	NDUFS1	S1		N	N
O	NDUFV2	V2		N	N
B	NDUFS8	S8		Q	Q
C	NDUFS7	S7		Q	Q
E	NDUFA6	A6		Q	Q
G	NDUFAB1	AB1		Q	Q
H	NDUFA5	A5		Q	Q
I	NDUFA7	A7	M2	Q	Q
J	NDUFA9	A9		Q	Q
N	NDUFA12	A12		N	N
P	NDUFS3	S3		Q	Q
Q	NDUFS2	S2		Q	Q
T	NDUFS6	S6		N	N
S	NDUFA1	A1		P_P	P_P-a
U	NDUFA3	A3		P_P	P_P-a
W	NDUFA13	A13		P_P	P_P-a
h	NDUFS5	S5		P_P	P_P-b
j	MT-ND3	ND3	M3	P_P	P_P-b
k	MT-ND4L	ND4L		P_P	P_P-b
m	MT-ND6	ND6		P_P	P_P-b
s	MT-ND1	ND1		P_P	P_P-a
u	NDUFA8	A8		P_P	P_P-a
X	NDUFAB1	AB1		P_D	P_D-b
Y	NDUFB2	B2		P_D	P_D-b
Z	NDUFB3	B3		P_D	P_D-b
a	NDUFB5	B5		P_D	P_D-a
b	NDUFB6	B6		P_D	P_D-a
c	NDUFB8	B8		P_D	P_D-b
d	NDUFB10	B10		P_D	P_D-a
e	NDUFB11	B11	M4	P_D	P_D-a
l	MT-ND5	ND5		P_D	P_D-b
n	NDUFB1	B1		P_D	P_D-a
o	NDUFB4	B4		P_D	P_D-a
p	NDUFB9	B9		P_D	P_D-b
r	MT-ND4	ND4		P_D	P_D-a
v	NDUFB7	B7		P_D	P_D-b
V	NDUFA11	A11		P_P	P_P-b
f	NDUFC1	C1		P_P	P_P-b
g	NDUFC2	C2	M5	P_P	P_P-b
i	MT-ND2	ND2		P_P	P_P-b
w	NDUFA10	A10		P_P	P_P-b

Table S3. Protein chain IDs and their associated numbers of the group II chaperonin TRiC/CCT in an open conformation, PDB ID 7YLV (Han et al., 2023), and the respective affiliation with structural modules, see Figure 2 in the original paper.

Chain name	Number	Structural module
A	1	M1
D	4	
G	3	
Z	6	
a	1	M2
d	4	
g	3	
z	6	
P	-	
B	2	M3
E	5	
H	7	
Q	8	
b	2	M4
e	5	
h	7	
q	8	

Table S4. Protein chain IDs and their associated numbers of the group II chaperonin TRiC/CCT in a closed conformation, PDB ID 7YLX (Han et al., 2023), and the respective affiliation with structural modules, see Figure 2 in the original paper.

Chain name	Number	Structural module
A	1	M1
G	3	
Z	6	
a	1	M2
g	3	
z	6	
B	2	M3
D	4	
E	5	
H	7	
Q	8	
b	2	M4
d	4	
e	5	
h	7	
q	8	
P	-	

Table S5. Protein chain IDs of the head of the bacteriophage Φ29, PDB ID 6QYD (Xu et al., 2019), and the respective affiliation with structural modules, see Figure 3 in the original paper.

Chain names	Structural module
1A, 1B, 1C, 1D, 1E, 1F, 2A, 2B, 2C, 2D, 2E, 3A, 3B, 3C, 3D, 3E, 3F, 4A, 4B, 4C, 4D, 4E, 4F, 1e, 1f, 2Z, 2a, 2b, 2c, 2d, 3e, 3f, 4e, 4f, 1k, 1l, 3k, 3l	M1
5A, 5B, 5C, 5D, 5E, 5F, 6A, 6B, 6C, 6D, 6E, 6F, 7A, 7B, 7C, 7D, 7E, 8A, 8B, 8C, 8D, 8E, 8F, 5e, 5f, 6e, 6g, 6h, 7Z, 7b, 7d, 8i, 8j, 7f, 6v	M2
7a, 6u, 9Q	M3
7c, 8f, 9P	M4
9A, 9B, 9C, 9D, 9E, 8e, 8g, 9F, 8k, 8m, 9G, 8q, 8s, 9H, 8w, 8y, 9I, 9M, 9O, 9J	M5
1G, 1H, 1I, 1J, 1K, 1L, 2F, 2G, 2H, 2I, 2J, 3G, 3H, 3I, 3J, 3K, 3L, 4G, 4H, 4I, 4J, 4K, 4L, 1i, 1j, 2e, 2f, 2g, 2h, 2i, 3i, 3j, 4g, 4h, 1o, 1p, 3o, 3p	M6
5G, 5H, 5I, 5J, 5K, 5L, 6G, 6H, 6I, 6J, 6K, 6L, 7F, 7G, 7H, 7I, 7J, 8G, 8H, 8I, 8J, 8K, 8L, 6f, 8h, 5g, 5h, 6k, 6l, 7e, 7g, 7h, 7i, 8l, 8p	M7
1M, 1N, 1O, 1P, 1Q, 1R, 2K, 2L, 2M, 2N, 2O, 3M, 3N, 3O, 3P, 3Q, 3R, 4M, 4N, 4O, 4P, 4Q, 4R, 1m, 1n, 2j, 2k, 2l, 2m, 2n, 3m, 3n, 4i, 4j, 1s, 1t, 3s, 3t	M8
5M, 5N, 5O, 5P, 5Q, 5R, 6M, 6N, 6O, 6P, 6Q, 6R, 7K, 7L, 7M, 7N, 7O, 8M, 8N, 8O, 8P, 8Q, 8R, 6i, 6j, 8n, 8o, 5i, 5j, 6o, 6p, 7j, 7k, 7l, 7m, 7n, 8r, 8v	M9
1S, 1T, 1U, 1V, 1W, 1X, 2P, 2Q, 2R, 2S, 2T, 3S, 3T, 3U, 3V, 3W, 3X, 4S, 4T, 4U, 4V, 4W, 4X, 1q, 1r, 2o, 2p, 2q, 2r, 2s, 3q, 3r, 4k, 4l, 1w, 1x, 3w, 3x	M10
5S, 5T, 5U, 5V, 5W, 5X, 6S, 6T, 6U, 6V, 6W, 6X, 7P, 7Q, 7R, 7S, 7T, 8S, 8T, 8U, 8V, 8W, 8X, 6m, 6n, 8t, 8u, 5k, 5l, 6s, 6t, 7o, 7p, 7q, 7r, 7s, 8x, 9L	M11
1Y, 1Z, 1a, 1b, 1c, 1d, 2U, 2V, 2W, 2X, 2Y, 3Y, 3Z, 3a, 3b, 3c, 3d, 4Y, 4Z, 4a, 4b, 4c, 4d, 1g, 1h, 3g, 3h, 1u, 1v, 2t, 2u, 2v, 2w, 2x, 3u, 3v, 4m, 4n	M12
5Y, 5Z, 5a, 5b, 5c, 5d, 6Y, 6Z, 6a, 6b, 6c, 6d, 7U, 7V, 7W, 7X, 7Y, 8Y, 8Z, 8a, 8b, 8c, 8d, 6q, 6r, 8z, 9K, 5m, 5n, 6w, 6x, 7t, 7u, 7v, 7w, 7x, 9N, 9R	M13

References

U. Brandt. Energy converting NADH: Quinone oxidoreductase (Complex I). *Annu. Rev. Biochem.*, 75:69–92, 2006.

S. Guerrero-Castillo et al. The assembly pathway of mitochondrial respiratory chain complex I. *Cell Metab.*, 25:128–139, 2017.

R. Guo et al. Architecture of human mitochondrial respiratory megacomplex I2III2IV2. *Cell*, 170:1247–1257.e12, 2017.

W. Han et al. Structural basis of plp2-mediated cytoskeletal protein folding by TRiC/CCT. *Sci. Adv.*, 9, 2023.

C. Hunte, V. Zickermann, and U. Brandt. Functional modules and structural basis of conformational coupling in mitochondrial complex I. *Science*, 329:448–451, 2010.

D. A. Stroud et al. Accessory subunits are integral for assembly and function of human mitochondrial complex I. *Nature*, 538:123–126, 2016.

J. Xu et al. Structural assembly of the tailed bacteriophage ϕ29. *Nat. Commun.*, 10, 2019.
